# UVA Radiation Enhances Lomefloxacin-Mediated Cytotoxic, Growth-Inhibitory and Pro-Apoptotic Effect in Human Melanoma Cells through Excessive Reactive Oxygen Species Generation

**DOI:** 10.3390/ijms21238937

**Published:** 2020-11-25

**Authors:** Artur Beberok, Zuzanna Rzepka, Jakub Rok, Klaudia Banach, Dorota Wrześniok

**Affiliations:** Department of Pharmaceutical Chemistry, Faculty of Pharmaceutical Sciences in Sosnowiec, Medical University of Silesia, Jagiellońska 4, 41-200 Sosnowiec, Poland; zrzepka@sum.edu.pl (Z.R.); jrok@sum.edu.pl (J.R.); d200667@365.sum.edu.pl (K.B.); dwrzesniok@sum.edu.pl (D.W.)

**Keywords:** lomefloxacin, UVA radiation, melanoma, oxidative stress, apoptosis

## Abstract

Melanoma, the most dangerous type of cutaneous neoplasia, contributes to about 75% of all skin cancer-related deaths. Thus, searching for new melanoma treatment options is an important field of study. The current study was designed to assess whether the condition of mild and low-dose UVA radiation augments the lomefloxacin-mediated cytotoxic, growth-inhibitory and pro-apoptotic effect of the drug in melanoma cancer cells through excessive oxidative stress generation. C32 amelanotic and COLO829 melanotic (BRAF-mutant) melanoma cell lines were used as an experimental model system. The combined exposure of cells to both lomefloxacin and UVA irradiation caused higher alterations of redox signalling pathways, as shown by intracellular reactive oxygen species overproduction and endogenous glutathione depletion when compared to non-irradiated but lomefloxacin-treated melanoma cells. The obtained results also showed that lomefloxacin decreased both C32 and COLO829 cells’ viability in a concentration-dependent manner. This effect significantly intensified when melanoma cells were exposed to UVA irradiation and the drug. For melanoma cells exposed to lomefloxacin or lomefloxacin co-treatment with UVA irradiation, the concentrations of the drug that decreased the cells’ viability by 50% (EC_50_) were found to be 0.97, 0.17, 1.01, 0.18 mM, respectively. Moreover, we found that the redox imbalance, mitochondrial membrane potential breakdown, induction of DNA fragmentation, and changes in the melanoma cells’ cell cycle distribution (including G_2_/M, S as well as Sub-G_1_-phase blockade) were lomefloxacin in a dose-dependent manner and were significantly augmented by UVA radiation. This is the first experimental work that assesses the impact of excessive reactive oxygen species generation upon UVA radiation exposure on lomefloxacin-mediated cytotoxic, growth-inhibitory and pro-apoptotic effects towards human melanoma cells, indicating the possibility of the usage of this drug in the photochemotherapy of malignant melanoma as an innovative medical treatment option which could improve the effectiveness of therapy. The obtained results also revealed that the redox imbalance intensification mediated by the phototoxic potential of fluoroquinolones may be considered as a more efficient treatment model of malignant melanoma and may constitute the basis for the development of new compounds with a high ability to excessive oxidative stress generation upon UVA radiation in cancer cells.

## 1. Introduction

The morbidity of malignant melanoma, the most aggressive form of skin cancer, is increasing globally. Moreover, a huge problem is the inefficiency of the current melanoma treatment options [1]. Melanoma derives from melanocytes—melanin-producing cells [2]. These cells exert pro-survival mechanisms that counteract harmful factors, including UV radiation [3]. The complex mechanisms for preventing cell death existing in melanocytes are further extended in melanoma cells, contributing to the melanoma pro-survival phenotype [2]. Amelanotic melanoma is a rare subtype of skin cancer that is characterised by the lack of melanin [4]. Because of its atypical presentation and delayed diagnosis, it has a poorer prognosis than pigmented melanoma subtypes. For unclear reasons, the prognosis among the patients with amelanotic metastases is worse than in the case of the pigmented ones [5]. In most cases, even the application of novel, targeted drugs (e.g., ipilimumab, nivolumab, vemurafenib) is not able to prolong the life of melanoma patients with advanced metastasis [6,7]. Thus, there is a need to discover novel therapeutic pathways that could ameliorate the efficacy of the therapy.

Lomefloxacin is widely used to treat infections of the respiratory or urinary tract. It belongs to fluoroquinolone derivatives—a group of synthetic antibiotics active against a broad spectrum of bacteria. Fluroquinolones (FQs) inhibit topisomerase II (gyrase DNA) and topoisomerase IV—enzymes involved in the replication and repair of bacterial DNA [8,9]. FQs antibiotics, as candidates for drug repositioning, are tested for potential anticancer activity. Nevertheless, scientific literature describes only a few cases of the potential use of lomefloxacin as an anti-cancer drug [10]. Previously, we have demonstrated that, as a result of the modulatory effect of the drug on the cell cycle distribution and the induction of apoptosis, lomefloxacin exerts cytotoxic and anti-proliferative effects on melanotic COLO829 melanoma cells [11].

Lomefloxacin exerts a relatively high phototoxic potential, causing severe adverse phototoxic reactions on pigment-containing tissues. The phototoxicity may occur as a result of FQ photodegradation and the molecules’ capacity to generate free oxygen radicals [12]. The halogenation of position 8 with the simultaneous fluorination of position 6 of the quinolone ring has significant phototoxic potential [13]. UVA-induced defluorination at the 8-position of the FQ molecule results in the generation of a reactive carbene intermediate, which in the presence of water and oxygen is converted to hydrogen peroxide with a subsequent production of hydroxyl radical via Fenton chemistry [12]. The heterolysis of the C8-F bond produces a triplet aryl cation which can react with DNA nucleotides, mainly purine bases [14,15,16], and this reaction has been proposed to be involved in the genotoxic effect of lomefloxacin. It is also known that reactive oxygen species (ROS) play an important role in apoptosis activation by many drugs and radiation treatments [17]. The agents inducing a high oxidative stress threshold in cancer cells may selectively target tumour cells [18,19]. Therefore, it should be taken into consideration that these findings concerning the ROS participation in the apoptosis induction of photosensitising agents may be useful for the application of photosensitisation as a cancer treatment option. Recently, berberine has been reported to be a photosensitive drug with possible use in photodynamic therapy (PDT), where the photosensitive drug is activated upon exposure to UVA radiation, causing massive DNA strand breaks in tumour cells [20].

There is a lack of data demonstrating the role of oxidative stress in the molecular mechanisms that are involved in both UVA and lomefloxacin-mediated photototoxic and pro-apoptotic effects towards melanoma cells. Therefore, in order to demonstrate the signalling pathways underlying cellular and molecular evidence for the role of UVA radiation in lomefloxacin-mediated anti-melanoma effects, the present study was designed to show whether the observed decrease in cell viability, cell cycle arrest, as well as apoptosis induction may be associated with excessive ROS generation. It was shown that BRAF mutant melanoma cells may display increased oxidative metabolism and increased dependency on mitochondria for survival [21]. With regard to the above considerations, both BRAF-mutant amelanotic C32 (characterised by a higher malignancy) and melanotic COLO829 melanoma cell lines were used as an in vitro model.

## 2. Results and Discussion

### 2.1. Low-Dose Uva Enhances the Cytotoxic and Growth-Inhibitory Effect of Lomefloxacin towards C32 and Colo829 Cells

**Figure 1:**
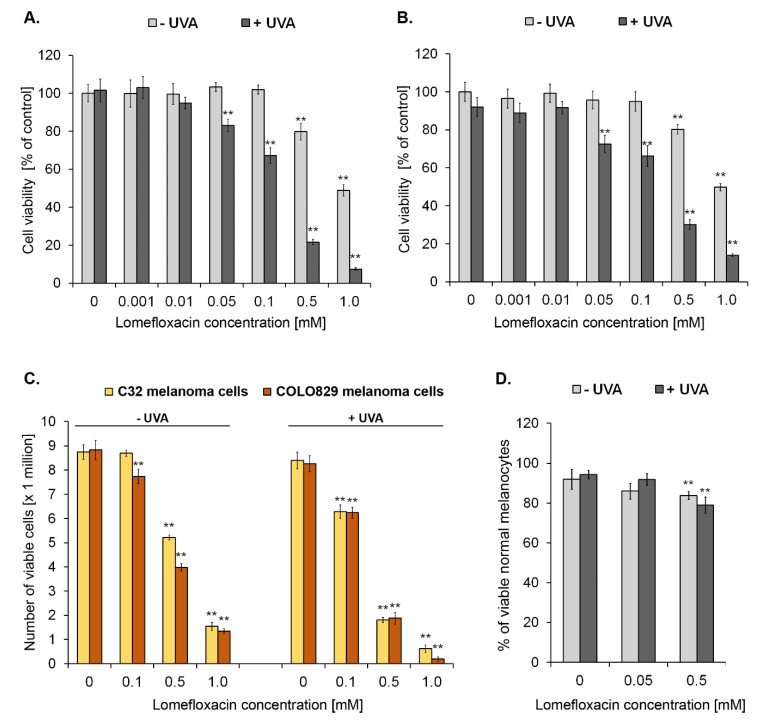
Simultaneous exposure of amelanotic C32 (**A**) and melanotic COLO829 (**B**) melanoma cells to lomefloxacin and UVA irradiation enhances the decrease in the viability of cells. The cells were pre-treated with lomefloxacin at the concentration range from 0.001 to 1.0 mM alone for 24 h or exposed to the drug and UVA irradiation (1.3 J/cm^2^). Mean values ± SEM from three independent experiments (*n* = 9) performed in triplicate are presented. ** *p* < 0.005 vs. control samples. Growth inhibitory effect of lomefloxacin and lomefloxacin co-treatment with UVA irradiation towards melanoma cells (**C**) and melanocytes (**D**). C32 and COLO829 cells were pre-treated with lomefloxacin at concentrations of 0.1, 0.5, and 1.0 mM alone for 24 h or exposed to the drug and UVA irradiation (1.3 J/cm^2^). Melanocytes were pre-treated with lomefloxacin at concentrations of 0.05 and 0.5 mM alone for 24 h or exposed to the drug and UVA irradiation (1.3 J/cm^2^). Mean values ± SEM from three independent experiments (*n* = 9) performed in triplicate are presented. ** *p* < 0.005 vs. control samples.

Oxidative stress or redox homeostasis imbalance may cause cell transition from quiescent to proliferative status, growth arrest, or cell death depending on the duration and extent of the redox imbalance [22]. In order to examine the effect of redox imbalance generated by lomefloxacin combined with UVA irradiation on the melanoma cell viability, C32 as well as COLO829 cells were pre-treated with the drug at concentrations of 0.001, 0.01, 0.05, 0.1, 0.5, and 1.0 mM for 24 h and then exposed to UVA radiation. The viability of cells was determined 24 h after the irradiation. As shown in Figure 1A,B, the exposure of cells to UVA irradiation alone had no significant effect on cell viability, pointing to the notion that at these exposure conditions the applied dosage of UVA radiation could be considered as a low and mild dose without any impact on the measured parameters. Following the lomefloxacin treatment, the drug only at the highest concentrations (0.5 mM and 1.0 mM) caused a significant decrease in the viability of both the tested melanoma cell lines by about 20% and 50%, respectively. Combined exposure to the drug at a concentration range from 0.05 to 1.0 mM and UVA irradiation significantly lowered the value of the tested parameter with the decrease in C32 and COLO829 viability by 17–93% and 28–86%, respectively. For melanoma cells exposed to lomefloxacin or lomefloxacin co-treatment with UVA irradiation, the concentrations of the drug that decreased the cells’ viability by 50% (EC_50_) were found to be 0.97, 0.17, and 1.01, and 0.18 mM, respectively. In our earlier study, it was reported that lomefloxacin alone decreased the COLO829 cells’ viability, with the values of the EC_50_ parameter established as 0.25 mM for a 72 h incubation time [11]. In the current study, the concentrations causing the 50% decrease in cell viability were significantly lower when the cells were pre-treated with lomefloxacin for 24 h and then exposed to UVA irradiation, pointing to the increased sensitivity of melanoma cells to lomefloxacin treatment when combined with UVA radiation.

To confirm the efficacy of lomefloxacin as well as lomefloxacin co-treatment with UVA irradiation on the cells’ growth inhibition, the fluorescence image cytometry technique was applied. As shown in Figure 1C, pre-treatment with lomefloxacin at concentrations of 0.5 and 1.0 mM inhibited the growth (expressed as the percentages of live cells) of both C32 and COLO829 melanoma cells by 40%, 83%, 55%, and 85%, respectively, as compared with the controls. The more marked decrease in this parameter was stated following the exposure of melanoma cells to all studied lomefloxacin concentrations (0.1, 0.5, and 1.0 mM) when combined with UVA irradiation—by 28%, 80%, and 93% (for C32 cells) and by 28%, 81%, and 98% (for COLO829 cells), respectively, showing the increased sensitivity of melanoma cells to the use of lomefloxacin and UVA radiation in combination. In our recent study [23], we have pointed to the fact that another fluoroquinolone antibiotic, ciprofloxacin, possess the ability to form complexes with MITF (in silico analysis), which promotes cell proliferation, has a pro-survival role in melanoma cells, and decreases its expression at the protein level (Western blot analysis), characterising the role of MITF protein in the anti-proliferative effect of the drug. Therefore, it may be concluded that the observed growth-inhibitory effect could be complex, and oxidative stress- and MITF-dependent.

In order to assess the selectivity in the mode of lomefloxacin co-treatment with UVA radiation action, the viability of human epidermal melanocytes was tested. The analysis was based on the non-fixed cell staining with acridine orange (total population) and DAPI (dead cells). The cytometric analysis revealed that lomefloxacin in combination with UVA radiation affected the viability of the tested normal cells less, since a decrease of ca. 20% in the percentages of viable normal melanocytes exposed to lomefloxacin at the concentration of 0.5 mM with UVA irradiation was observed (Figure 1D).

**Figure 2:**
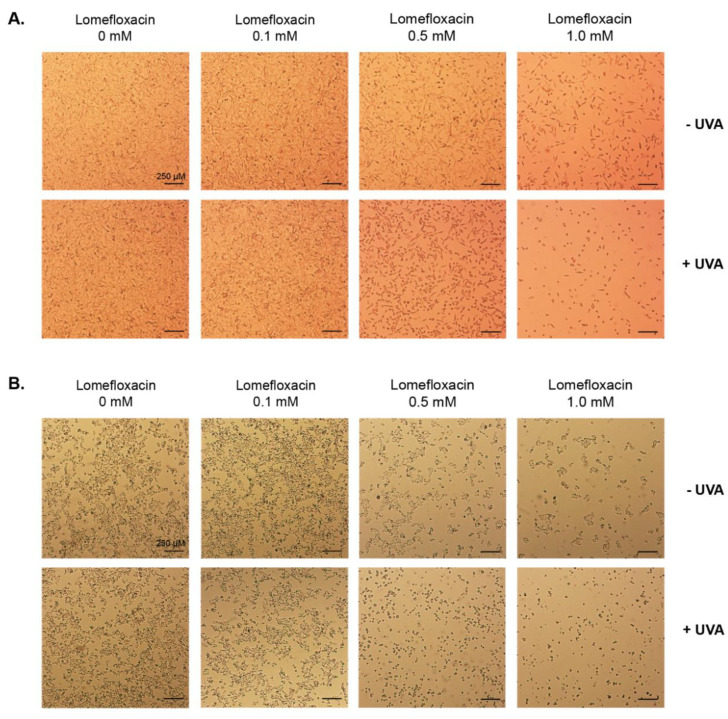
Combined treatment of C32 (**A**) and COLO829 (**B**) cells with lomefloxacin and UVA irradiation augments the induction of apoptotic features in melanoma cells. The cells were pre-treated with lomefloxacin at concentrations of 0.1, 0.5, and 1.0 mM alone for 24 h or exposed to the drug and UVA irradiation (1.3 J/cm^2^) and observed under a light inverted microscope at a 40x magnification (scale bar 250 µm).

The morphology of melanoma cells was assessed by the use of a light inverted microscope at a 40× magnification (Figure 2). The untreated (control) C32 and COLO829 cells exerted adherent growth, cell–cell cohesion, and characteristic shape. The pre-treatment of cells with lomefloxacin alone in concentrations of 1.0 mM for 24 h resulted in apoptotic features (rounding, shrinkage, and loss of cell–cell contact). Moreover, it has been demonstrated that the co-treatment with lomefloxacin at concentrations of 0.5 and 1.0 mM and UVA irradiation augments the development of the apoptotic features in both studied melanoma cell lines. In all analysed samples (excluding 0.1 mM of lomefloxacin), a decrease in the cell number was also stated. Nakai et al. [24] assessed the induction of apoptosis in human promyelocytic leukemic cells by examining their morphology after incubation with lomefloxacin, UVA radiation, and lomefloxacin combined with UVA irradiation. At these exposure conditions, membrane blebbing and cell shrinkage were clearly seen only in the group treated with lomefloxacin and UVA irradiation, suggesting that the differences in the strength and mode of action of the tested fluoroquinolone derivative could be attributed to the difference in the origin of the cell type.

### 2.2. UVA Radiation Augments Lomefloxacin-Mediated Redox Imbalance in C32 and Colo829 Melanoma Cells

**Figure 3:**
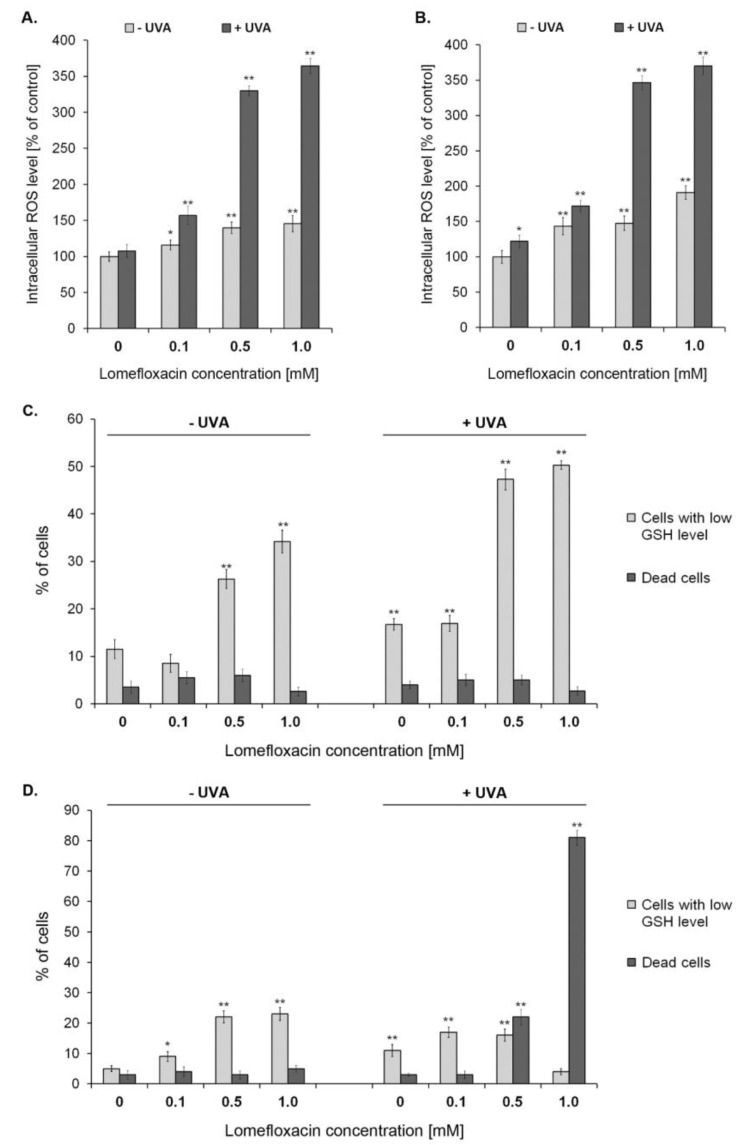
UVA radiation-intensified lomefloxacin induces ROS production in amelanotic C32 (**A**) and melanotic COLO829 (**B**) melanoma cells. The cells were pre-treated with lomefloxacin at concentrations of 0.1, 0.5, and 1.0 mM alone for 24 h or exposed to the drug and UVA irradiation (1.3 J/cm^2^). The data are expressed as percentages of the controls normalised to a number of living cells. Mean values ± SEM from three independent experiments (*n* = 9) performed in triplicate are presented. * *p* < 0.05, ** *p* < 0.005 vs. control samples. Co-treatment of C32 (**C**) and COLO829 (**D**) melanoma cells with lomefloxacin and UVA irradiation augments cellular GSH depletion. The cells were pre-treated with lomefloxacin at concentrations of 0.1, 0.5, and 1.0 mM alone for 24 h or exposed to the drug and UVA irradiation (1.3 J/cm^2^). Mean values ± SEM from three independent experiments (*n* = 9) performed in triplicate are presented. ** *p* < 0.005 vs. control samples. Representative histograms are available in the Supplementary Materials S1.

H_2_DCFDA staining was used to detect ROS overproduction in C32 as well as COLO829 melanoma cells exposed to lomefloxacin or the drug combined with UVA irradiation. As shown in Figure 3A,B, the exposure of C32 as well as COLO829 melanoma cells to lomefloxacin alone or UVA radiation combined with the 24 h drug pretreatment leads to ROS overproduction in a concentration-dependent manner. The treatment of cells with lomefloxacin at concentrations of 0.1, 0.5, and 1.0 mM, but without UVA radiation, enhanced ROS production by 16%, 39%, and 46% for C32 cells or 43%, 47%, and 91% for COLO829 cells, respectively, in comparison to the untreated cells (controls). This effect significantly intensified when the studied melanoma cells were simultaneously exposed to UVA radiation and the drug at concentrations of 0.1, 0.5, and 1.0 mM. At these exposure conditions, the ROS generation increased by 57%, 230%, and 264% for amelanotic C32 cells or 72%, 246%, and 270% for melanotic COLO829 cells, respectively. When compared the two analysed in vitro models, it could be stated that the intracellular ROS levels after the drug with or without UVA irradiation were higher in the case of melanotic melanoma cells, indicating the greater capacity of lomefloxacin, at the tested exposure conditions, to induce redox imbalance in COLO829 cells. Previously, we have demonstrated that lomefloxacin without the combined exposure of melanotic melanoma cells to UVA radiation after a 24 h period of incubation significantly enhanced the level of ROS [11]. These findings, strengthened by the data concerning the photodynamically induced ROS overproduction in C32 as well as COLO829 after lomefloxacin treatment, may suggest that the drug is able to alter the intracellular redox status of melanoma cells via generating excessive ROS.

Tumour cells can be sensitised to antitumor therapy by disabling antioxidant defences—e.g., through metabolic inhibition, as was demonstrated for nicotinamide adenine dinucleotide phosphate (NADPH) or glutathione. Thus, strategies aimed at altering redox signalling in cancer cells and intended to disable key antioxidant systems in the presence of ROS enhancers could represent promising new anticancer treatments [22]. The tripeptide, glutathione, is a key intracellular thiol. It is predominantly present in cells in the reduced form (GSH). Under oxidising conditions, the oxidation of GSH to its disulphide (GSSG) results in a decreased GSH-to-GSSG ratio. GSH has a crucial role in maintaining redox homeostasis, which is essential for apoptosis, cell differentiation, and proliferation. GSH interacts with ROS and is necessary for the action of GSH peroxidases and GSH-S-transferases. It is also involved in the redox regulation of protein thiols [25]. A shift in the cellular GSH-to-GSSG balance in favour of the GSSG (caused by direct GSH oxidation or GSH extrusion from cells) has been reported to be an early event of apoptosis [26,27,28].

In the current study, it was observed that the pre-treatment of melanoma cells with the drug at concentrations of 0.5 and 1.0 mM for 24 h increased the percentages of PI-negative/VB-48^TM^-negative cells exhibiting low levels of GSH from 12% and 5% (controls) to 26% and 34% for C32 cells (Figure 3C,D and Figure S1) and 22% and 23%, for COLO829 cells (Figure 3), respectively. Interestingly, following the combined exposure of cells to lomefloxacin at concentrations of 0.5 and 1.0 mM and UVA irradiation, the GSH depletion intensified significantly only in the case of amelanotic C32 melanoma cells. Under these conditions, about a three-fold increase in the percentages of cells exhibiting low levels of GSH was seen when compared to the controls, pointing to the capacity of lomefloxacin to cause the UVA-mediated disruption of the redox balance, especially in C32 melanoma cells. In the case of COLO829 cells, a significant increase in the percentages of PI-positive (dead cells) after drug exposure with UVA irradiation was noticed, from 3% (controls) to about 20% and 80% (lomefloxacin at concentrations 0.5 and 1.0 mM, respectively), indicating that lomefloxacin and UVA radiation-induced GSH depletion could be considered as an early mechanism underlying the drug pro-apoptotic effect on melanotic melanoma cells.

### 2.3. The Usage of Lomefloxacin and Uva Radiation in Combination Intensifies the Pro-Apoptotic Response of Melanoma Cells

High intracellular ROS levels may contribute to the opening of the mitochondrial permeability transition pore and the release of cytochrome c to the cytosol, which is followed by the formation of apoptosome and caspase activation [29]. The opening of the mitochondrial permeability transition pore is also facilitated by the direct depletion of the mitochondrial GSH [30]. Thus, GSH has an important role in the regulation of the intrinsic apoptotic cascade [29].

**Figure 4:**
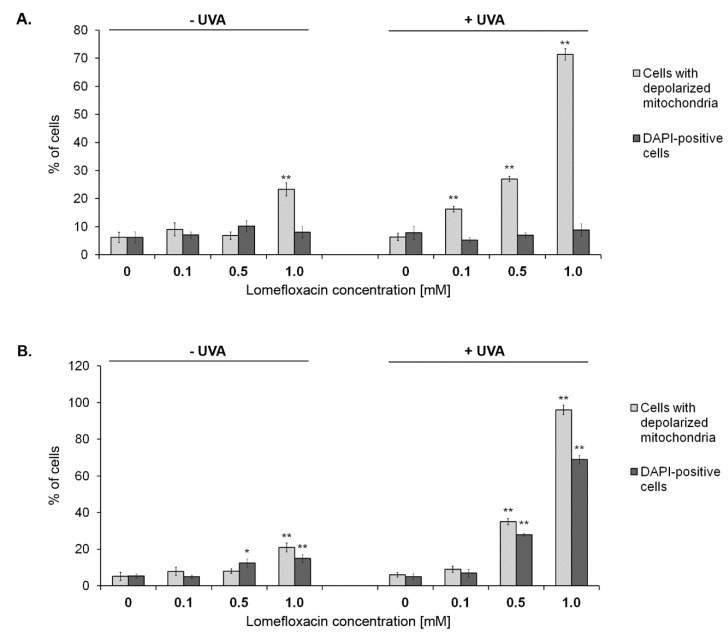
Cytometric analysis of the mitochondrial transmembrane potential disruption in C32 (**A**) and COLO829 (**B**) melanoma cells exposed to lomefloxacin or lomefloxacin in combination with UVA irradiation. The cells were pre-treated with lomefloxacin at concentrations of 0.1, 0.5, and 1.0 mM alone for 24 h or exposed to the drug and UVA irradiation (1.3 J/cm^2^). Mean values ± SEM from three independent experiments (*n* = 9) performed in triplicate are presented. * *p* < 0.05, ** *p* < 0.005 vs. control samples. Representative histograms are available in the Supplementary Materials S2.

In order to confirm the mitochondria involvement in the apoptotic cell death pathway under the tested exposure conditions, the mitochondrial depolarisation in C32 and COLO829 melanoma cells was estimated. Following the image cytometric analysis, the increase in the percentages of mitochondrial membrane depolarised C32 (Figure 6) and COLO829 (Figure 4 and Figure S2) cells exposed to lomefloxacin alone was only seen when the cells were exposed to the highest drug concentration (1.0 mM). At this exposure condition, the percentages of cells with the permeabilisation of the outer membrane of mitochondria increased and was determined to be about 20%, while the value determined for the controls was 6% and 5%, respectively. This effect was more marked in the combined treatment model and was detected for all the studied lomefloxacin concentrations (excluding the drug at a concentration of 0.1 mM in parallel with COLO829 cells), with the percentages of mitochondrial membrane depolarised cells increased from about 6% (controls) to 16%, 27%, and 71% for C32 cells as well as to 35% and 96% for COLO829 cells, respectively, which indicates that GSH depletion mediated by excessive ROS formation is essential in cellular susceptibility to apoptosis.

Interestingly, a significant increase in the blue DAPI fluorescence was observed only following the exposure of COLO829 cells to lomefloxacin (24 h pre-treatment) and lomefloxacin (24 h pre-treatment) combined with UVA irradiation (Figure 4 and Figure S2). For the tested cell line, this effect was noticed especially after the lomefloxacin co-treatment with UVA irradiation, where the percentages of late apoptotic DAPI-positive cells increased from 5% (control) to 28% and 69% for the drug at concentrations of 0.5 and 1.0 mM, respectively, showing the greater sensitivity of COLO829 cells to the tested apoptotic stimuli.

**Figure 5:**
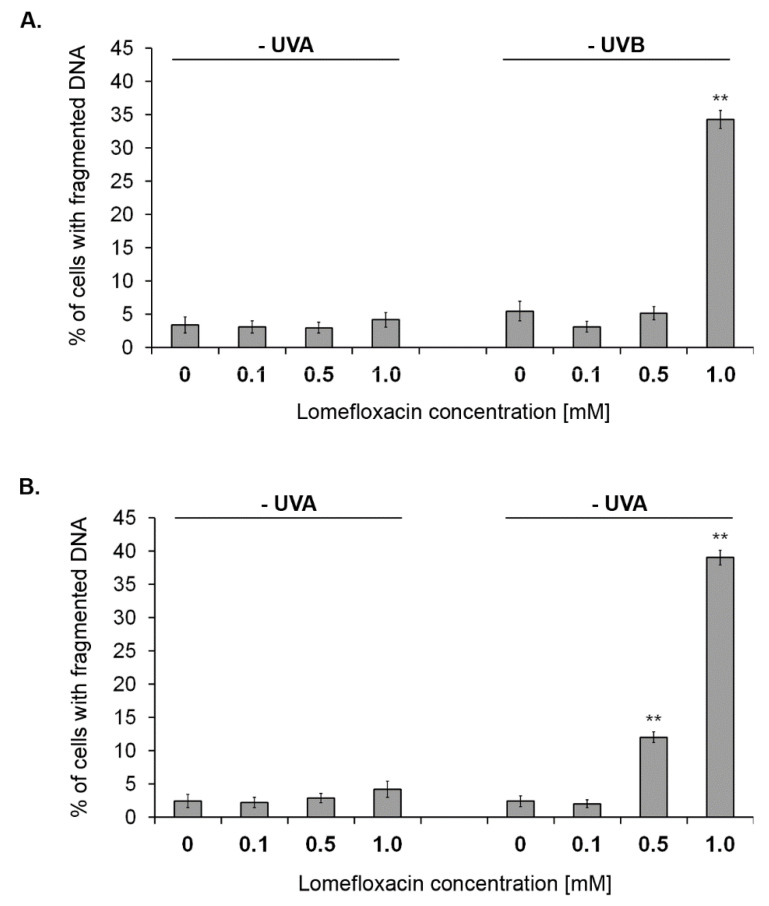
UVA radiation-intensified lomefloxacin induces DNA fragmentation in amelanotic C32 (**A**) and melanotic COLO829 (**B**) melanoma cells. Melanoma cells were pre-treated with lomefloxacin at concentrations of 0.1, 0.5, and 1.0 mM alone for 24 h or exposed to the drug and UVA irradiation (1.3 J/cm^2^). The data are expressed as percentages of the controls. Mean values ± SEM from three independent experiments (*n* = 9) performed in triplicate are presented. ** *p* < 0.005 vs. control samples. Representative histograms are available in the Supplementary Materials S3.

Oxidative stress may cause single- and double-strand DNA fragmentation and may be associated with permeability alterations and facilitate the release of death-related molecular signals [29]. The cleavage of the chromosomal DNA is an integral part of apoptosis and is a key apoptotic marker [29]. To further explore the ROS-mediated apoptosis intensification in C32 and COLO829 melanoma cells, in the present study the effect of lomefloxacin under the tested exposure conditions on the DNA fragmentation was analysed with the use of image cytometry (Figure 5 and Figure S3). Although the treatment of cells with UVA irradiation or lomefloxacin alone had no effect on this apoptotic marker, the induction of DNA fragmentation in amelanotic C32 and melanotic COLO829 melanoma cells was noticed after combined treatment with lomefloxacin and UVA irradiation. The co-treatment of C32 and COLO829 cells with the drug at a concentration of 1.0 mM under exposure to UVA radiation increased the percentages of population of cells with fragmented DNA from 3% and 2% (controls) to 34% and 39%, respectively. Interestingly, in the case of COLO829 cells the six-fold increase in this parameter was also noticed for lower lomefloxacin concentration (0.5 mM), confirming the acquired data, which indicates (i) the fundamental role of excessive oxidative stress generation upon UVA radiation in the signalling pathway underlying lomefloxacin-mediated pro-apoptotic effect, (ii) the greater sensitivity of melanotic COLO829 melanoma cells (characterised by higher melanin biopolymer content) to apoptotic stimuli (in the form of combined treatment with lomefloxacin and UVA radiation) as a result of the stated higher redox imbalance.

### 2.4. Effect of Co-Treatment with Lomefloxacin and Uva Irradiation on Cell-Cycle Distribution of C32 and Colo829 Melanoma Cells

**Figure 6:**
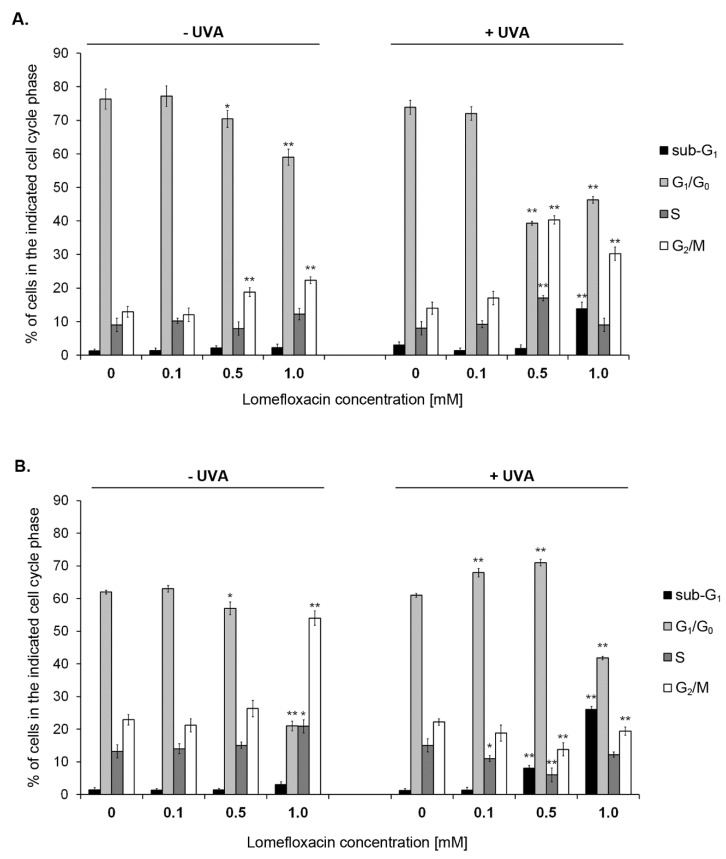
Changes in the cell cycle distribution of C32 (**A**) and COLO829 (**B**) melanoma cells after being cultured in the presence of lomefloxacin and UVA irradiation. Melanoma cells were pre-treated with lomefloxacin at concentrations of 0.1, 0.5, and 1.0 mM alone for 24 h or exposed to the drug and UVA irradiation (1.3 J/cm^2^). The data are expressed as percentages of the controls. Mean values ± SEM from three independent experiments (*n* = 9) performed in triplicate are presented. * *p* < 0.05, ** *p* < 0.005 vs. control samples. Representative histograms are available in the Supplementary Materials S4.

To explore the mode of action responsible for the growth inhibition of the melanoma cells treated with lomefloxacin and lomefloxacin combined with UVA irradiation, the cell cycle analysis was assessed using image cytometry and DAPI staining. The cells were distributed among four major phases of the cell cycle: Sub-G_1_, G_1_/G_0_, S, and G_2_/M phase. As shown in Figure 6, the pre-treatment of amelanotic C32 cells with lomefloxacin at concentrations 0.5 and 1.0 mM contributed to the G_2_/M phase arrest, with the percentages of G_2_/M fraction increased from 13% (control) to 19% and 22%, respectively. This effect significantly intensified upon the simultaneous exposure of cells to the drug and UVA irradiation, causing the 2- to 3-fold increase among the population of cells accumulated in the G_2_/M phase. In the case of COLO829 melanoma cells, the G_2_/M phase block was noticed only for non-irradiated but lomefloxacin (1.0 mM)-exposed cells (Figure 6 and Figure S4). At these exposure conditions, the percentages of G_2_/M fraction increased from 22% (control) to 54%. The co-treatment of COLO829 cells with the drug in the highest concentration (1.0 mM) elevated the population of cells arrested in the Sub-G_1_ phase of the cell-cycle from 1% (control) to 26%. A similar effect was seen in the case of C32 cells with the increase in the analysed parameter from 2% to 14%, respectively. Moreover, the increase in the percentage of cells in S phase was observed for C32 cells co-treated with lomefloxacin at a concentration of 0.5 mM and UVA irradiation, as well as for non-irradiated COLO829 cells incubated with lomefloxacin at a concentration of 1.0 mM. This effect may result from the activation of the intra-S checkpoint and, thus, the slowing of replication in response to DNA damage [31]. Previously, we have demonstrated that the exposure of COLO829 melanoma cells to lomefloxacin at concentrations of 0.1 and 1.0 mM for 24 and 48 h did not affect the Sub-G_1_ phase block [11], pointing to the significant role of oxidative stress overproduction in the sensitivity of the melanoma cell response to the growth inhibitory and pro-apoptotic effect of the drug observed when combined with UVA radiation.

## 3. Conclusions

The presented work is a part of complex and multi-directional design study targeted at fluoroquinolone antibiotics repurposing in malignant melanoma chemotherapy. Previously, we have demonstrated that, as a result of the modulatory effect of the drug on the cell cycle distribution and the induction of apoptosis, lomefloxacin exerts cytotoxic and anti-proliferative effects on melanotic COLO829 melanoma cells [11]. Encouraged by the findings, the obtained results gave us directions for further, more meticulous studies. There is lack of data that demonstrate the role of excessive oxidative stress generation in the molecular mechanisms involved in both UVA and lomefloxacin-mediated photototoxic and pro-apoptotic effect towards melanoma cells. The data presented herein provide the first evidence that (i) the excessive production of ROS in melanoma cells stated under combined treatment with lomefloxacin and UVA irradiation intensified the loss of C32 and COLO829 cells’ viability, growth inhibition, and apoptosis intrinsic signalling pathway mediated by the mitochondria, indicating the possibility of the use of this drug in the photochemotherapy of malignant melanoma as an innovative medical treatment option which could improve the effectiveness of the therapy; (ii) the redox imbalance intensification mediated by the phototoxic potential of fluoroquinolone antibiotics, which may be considered as a more efficient treatment model of malignant melanoma, which may constitute the basis for the search of new compounds with a high ability of excessive oxidative stress generation upon UVA radiation in cancer cells.

## 4. Materials and Methods

### 4.1. Chemicals

Lomefloxacin hydrochloride was purchased from Sigma-Aldrich Inc. (St. Luis, MO, USA). Growth medium DMEM, RPMI 1640, as well as amphotericin B, penicillin, streptomycin, fetal bovine serum, and trypsin/EDTA were obtained from Cytogen (Zgierz, Poland). The growth medium M-254 as well as a human melanocyte growth supplement-2 (HMGS-2) were acquired from Cascade Biologics (Portland, OR, USA). Cell Proliferation Reagent WST-1 was purchased from Roche GmbH (Mannheim, Germany). Solutions: 3 (1 µg/mL DAPI, 0.1% triton X-100 in PBS), 5 (VB-48^TM^, propidium iodide—PI, acridine orange—AO), 7 (200 µg/mL JC-1), 8 (1 µg/mL DAPI in PBS), NC-Slide A2 and Via-1-Cassette (AO and DAPI fluorophores) were obtained from ChemoMetec (Lillerød, Denmark). The remaining chemicals were produced by Sigma-Aldrich Inc. (USA).

### 4.2. UVA Irradiation Procedure

The source of UVA radiation (λ_max_ = 365 nm) was a filtered lamp BVL-8.LM (Vilber Lourmat, France). The analysed cells were irradiated for 30 min at an intensity of 720 μW/cm^2^ which corresponds to a dose of 1.3 J/cm^2^. The irradiation was carried out after a 24 h treatment of cells with lomefloxacin. Simultaneously, the non-irradiated cell cultures were kept in the dark at 37 °C and 5% CO_2_. Before irradiation, the culture medium was replaced by PBS in all tested samples. After irradiation, the PBS was removed and melanoma cells were incubated in the growth medium for the next 24 h.

### 4.3. Cell Culture

BRAF-mutant human amelanotic C32 and melanotic COLO829 melanoma cell lines were obtained from ATCC (USA). C32 cells were cultured in high-glucose DMEM medium, whereas COLO829 cells were incubated in RPMI 1640 (with L-glutamine). Both media were supplemented with 10% fetal bovine serum, penicillin (100 U/mL), neomycin (10 μg/mL), and amphotericin B (0.25 µg/mL).

Human epidermal melanocytes (HEMn-DP) were purchased from Cascade Biologics, UK. The growth medium was supplemented with a human melanocyte growth supplement-2 (HMGS-2) as well as antibiotics: penicillin (100 U/mL), neomycin (10 μg/mL), and amphotericin B (0.25 μg/mL).

All the cultures were grown at 37 °C in 5% CO_2_. All the experiments were performed using cells in the passages 5–12.

### 4.4. H_2_DCFDA Cellular ROS Detection Assay

The oxidation of 2,7-dichlorodihydrofluorescein diacetate (H_2_DCFDA) into 2,7-dichlorofluorescein (DCF) was used to assess the ROS generation in C32 and COLO829 melanoma cells after combined treatment with lomefloxacin and UVA irradiation [11]. In brief, 2500 cells per well were placed in a 96-well dark microplate in a supplemented growth medium and incubated at 37 °C and 5% CO_2_ for 24 h. After incubation, the medium was removed and the cells were treated with lomefloxacin solutions ranging from 0.1 to 1.0 mM. Afterwards, the melanoma cells were exposed to UVA radiation according to the procedure. After 24 h, the medium was removed and the cells were incubated with 10 µM of H_2_DCFDA for 30 min at 37 °C and washed twice with PBS to remove excess dye. The fluorescence was read at wavelengths of 485 nm of excitation and 530 nm of emission using a microplate reader Infinite 200 Pro (Tecan, The Switzerland). The obtained results, normalised to a number of living cells, were finally expressed as a percentage of the controls.

### 4.5. Assessment of Intracellular Thiol Levels

The GSH levels in C32 and COLO829 cells were measured using the NucleoCounter NC-3000 (ChemoMetec, Lillerød, Denmark) fluorescence image cytometer following a previously reported protocol [32,33]. Cells were exposed to lomefloxacin at concentrations of 0.1, 0.5, and 1.0 mM for 24 h. Afterwards, the melanoma cells were exposed to UVA irradiation according to the procedure. Then, the samples were stained with solution 5 and analysed with the use of the NucleoView NC-3000 software (ChemoMetec, Denmark). The obtained histograms were used to demarcate the percentage of PI-negative cells with a low cellular GSH level, PI-negative cells with a high cellular GSH level (healthy cells), and PI-positive cells (dead cells).

### 4.6. Cell Viability

The viability of melanoma cells (C32 and COLO829) was evaluated by the WST-1-based microplate colorimetric assay following a previously described protocol [34]. In brief, 2500 cells per well were pre-incubated in DMEM or RPMI 1640 for 24 h. Subsequently, the medium was replaced with different lomefloxacin solutions (0.001, 0.01, 0.05, 0.1, 0.5, and 1.0 mM) and the cells were incubated with the drug for 24 h. Afterwards, the melanoma cells were exposed to UVA irradiation according to the procedure. WST-1 was added 3 h prior to the end of the incubation periods. The absorbance of samples was measured at 440 nm with a reference wavelength of 650 nm using a microplate reader Infinite 200 Pro (Tecan, Switzerland). The controls were normalised to 100% for each assay and treatments were expressed as the percentages of the controls.

### 4.7. Cell Count Assay

The cell count assay of the analysed melanoma cells was performed with the use of fluorescence image cytometry, which uses the cell stain acridine orange for cell detection and the nucleic acid stain DAPI for the detection of non-viable cells. The cells were exposed to lomefloxacin at concentrations of 0.1, 0.5, and 1.0 mM for 24 h. Afterwards, the melanoma cells were exposed to UVA irradiation according to the procedure. Samples of the cell suspensions were drawn directly into the Via1-Cassette^TM^ and analysed with the use of the NucleoView NC-3000 software (ChemoMetec, Denmark).

### 4.8. Cell Morphology Assessment

C32 and COLO829 melanoma cells were cultured in T-25 flasks (1 × 10^6^ cells/flask) in DMEM and RPMI 1640 supplemented medium, respectively. Pre-treatment with the drug at concentrations of 0.1, 0.5, and 1.0 mM began 24 h after seeding. Afterwards, the melanoma cells were exposed to UVA irradiation according to the procedure and analysed with the use of a light inverted microscope NIKON TS100F (Tokyo, Japan).

### 4.9. Mitochondrial Potential Assay

The mitochondrial transmembrane potential was measured using the NucleoCounter NC-3000 fluorescence image cytometer following a previously described method [32,33]. Melanoma cells were incubated for 24 h with lomefloxacin at concentrations of 0.1, 0.5, and 1.0 mM. Afterwards, the melanoma cells were exposed to UVA irradiation according to the procedure. Samples were stained with solutions 7 at 37 °C for 15 min. At the end of analysis, the cell pellets were resuspended in 250 µL solution 8 and analysed immediately using the NucleoView NC-3000 software (ChemoMetec, Denmark). The obtained scatter plots and histograms were used to demarcate the percentage of polarised/healthy cells, depolarised/apoptotic cells, and DAPI-positive/late apoptotic cells.

### 4.10. Cell Cycle-DAPI/DNA Fragmentation Assay

Cell cycle and DNA fragmentation analyses were performed with the use of image cytometry technique [35]. In brief, C32 and COLO829 melanoma cells were treated with different lomefloxacin concentrations (0.1, 0.5, and 1.0 mM) for 24 h. Afterwards, the melanoma cells were exposed to UVA irradiation according to the procedure. Cells were fixed with 70% cold-ethanol at 4 °C for 24 h, stained with solution 3 containing DAPI for 5 min at 37 °C and analysed using the NucleoView NC-3000 software (ChemoMetec, Lillerød, Denmark). The obtained DNA content histograms were used to demarcate cells in the different cell cycle stages or to point apoptotic cells with fragmented DNA having less than 1 DNA equivalent (so-called Sub-G_1_ cells).

### 4.11. Statistical Analysis

In all the in vitro experiments, the mean values of at least three separate experiments performed in triplicate (*n* = 9) ± standard error of the mean (SEM) were calculated. The results were analysed statistically using the GraphPad Prism 6.01 software by means of a two-way ANOVA as well as Dunnett’s comparison test. In all cases, statistical significance was found for *p*-value lower than 0.05.

## Supplementary Materials

Supplementary Materials can be found at https://www.mdpi.com/1422-0067/21/23/8937/s1. Figure S1. Co-treatment of C32 (**A**) and COLO829 (**B**) melanoma cells with lomefloxacin and UVA irradiation augments cellular GSH depletion. Histograms presenting the changes of GSH level in cells treated with lomefloxacin at concentrations of 0.1 mM, 0.5 mM and 1.0 mM alone or exposed to the drug and UVA-irradiation (1.3 J/cm^2^). The histograms are representative for three independent experiments with similar results. Legend: Q1ll—GSH-depleted cells; Q1ul—dead cells. Figure S2. The effect of combined treatment with lomefloxacin and UVA irradiation on permeabilization of the outer membrane of mitochondria in melanoma cells. Scatter plots presenting changes in JC-1 and DAPI intensity in C32 (**A**) and COLO829 (**B**) cells following 24-h pre-treatment with 0.1 mM, 0.5 mM and 1.0 mM lomefloxacin or exposed to the drug and UVA irradiation (1.3 J/cm2). The presented graphs are representative for three independent experiments. Legend: Q1ur—polarized (healthy) cells; Q1lr—depolarized (early apoptotic) cells; M1—DAPI-positive (late apoptotic) cells(B). Figure S3. UVA radiation intensified lomefloxacin-induces DNA fragmentation in amelanotic C32 (**A**) and melanotic COLO829 (**B**) melanoma cells. The histograms are representative for three independent experiments with similar results. The effect of lomefloxacin or lomefloxacin co-treatment with UVA irradiation on DNA fragmentation was investigated by fluorescence image cytometer after DAPI staining. Legend: M1—cells having less than 1 DNA equivalent (so-called sub-G_1_ cells); M2—cells having 1 or more than 1 DNA equivalent. Melanoma cells were pre-treated with lomefloxacin at concentrations of 0.1 mM, 0.5 mM and 1.0 mM alone for 24 h or exposed to the drug and UVA irradiation (1.3 J/cm^2^). Figure S4. Changes in the cell cycle distribution of C32 (**A**) and COLO829 (**B**) melanoma cells cultured in the presence of lomefloxacin and UVA irradiation. Histograms presenting the cell cycle analysis in cells exposed to lomefloxacin alone or in combination with UVA irradiation after DAPI staining. The presented histograms are representative for three independent experiments with similar results. Legend: M1—sub-G_1_ phase; M2—G_1_/G_0_ phase; M3—S phase; M4—G_2_/M phase. Melanoma cells were pre-treated with lomefloxacin at concentrations of 0.1 mM, 0.5 mM and 1.0 mM alone for 24 h or exposed to the drug and UVA irradiation (1.3 J/cm^2^).
