# Supplementary material for: UVA Radiation Enhances Lomefloxacin-Mediated Cytotoxic, Growth-Inhibitory and Pro-Apoptotic Effect in Human Melanoma Cells through Excessive Reactive Oxygen Species Generation
Source: Int J Mol Sci. 2020 Nov 25;21(23):8937. doi: 10.3390/ijms21238937 (PMC7728064; doi:10.3390/ijms21238937)
Supplement: Supplementary file 1 [file ijms-21-08937-s001.zip › Supplementary - captions.docx]

**S 1.** Co-treatment of C32 (**A**) and COLO829 (**B**) melanoma cells with lomefloxacin and UVA irradiation augments cellular GSH depletion. Histograms presenting the changes of GSH level in cells treated with lomefloxacin at concentrations of 0.1 mM, 0.5 mM and 1.0 mM alone or exposed to the drug and UVA-irradiation (1.3 J/cm^2^). The histograms are representative for three independent experiments with similar results. Legend: Q1ll – GSH-depleted cells; Q1ul – dead cells.

**S 2.** The effect of combined treatment with lomefloxacin and UVA irradiation on permeabilization of the outer membrane of mitochondria in melanoma cells. Scatter plots presenting changes in JC-1 and DAPI intensity in C32 (**A**) and COLO829 (**B**) cells following 24-h pre-treatment with 0.1 mM, 0.5 mM and 1.0 mM lomefloxacin or exposed to the drug and UVA irradiation (1.3 J/cm2). The presented graphs are representative for three independent experiments. Legend: Q1ur – polarized (healthy) cells; Q1lr – depolarized (early apoptotic) cells; M1 – DAPI-positive (late apoptotic) cells. (B)

**S 3.** UVA radiation intensified lomefloxacin-induces DNA fragmentation in amelanotic C32 (**A**) and melanotic COLO829 (**B**) melanoma cells. The histograms are representative for three independent experiments with similar results. The effect of lomefloxacin or lomefloxacin co-treatment with UVA irradiation on DNA fragmentation was investigated by fluorescence image cytometer after DAPI staining. Legend: M1 – cells having less than 1 DNA equivalent (so-called sub-G_1_ cells); M2 – cells having 1 or more than 1 DNA equivalent. Melanoma cells were pre-treated with lomefloxacin at concentrations of 0.1 mM, 0.5 mM and 1.0 mM alone for 24 h or exposed to the drug and UVA irradiation (1.3 J/cm^2^).

**S 4.** Changes in the cell cycle distribution of C32 (**A**) and COLO829 (**B**) melanoma cells cultured in the presence of lomefloxacin and UVA irradiation. Histograms presenting the cell cycle analysis in cells exposed to lomefloxacin alone or in combination with UVA irradiation after DAPI staining. The presented histograms are representative for three independent experiments with similar results. Legend: M1- sub-G_1_ phase; M2 – G_1_/G_0_ phase; M3 – S phase; M4 – G_2_/M phase. Melanoma cells were pre-treated with lomefloxacin at concentrations of 0.1 mM, 0.5 mM and 1.0 mM alone for 24 h or exposed to the drug and UVA irradiation (1.3 J/cm^2^).
