# Supplementary figures and images for: UVA Radiation Enhances Lomefloxacin-Mediated Cytotoxic, Growth-Inhibitory and Pro-Apoptotic Effect in Human Melanoma Cells through Excessive Reactive Oxygen Species Generation
Source: Int J Mol Sci. 2020 Nov 25;21(23):8937. doi: 10.3390/ijms21238937 (PMC7728064; doi:10.3390/ijms21238937)

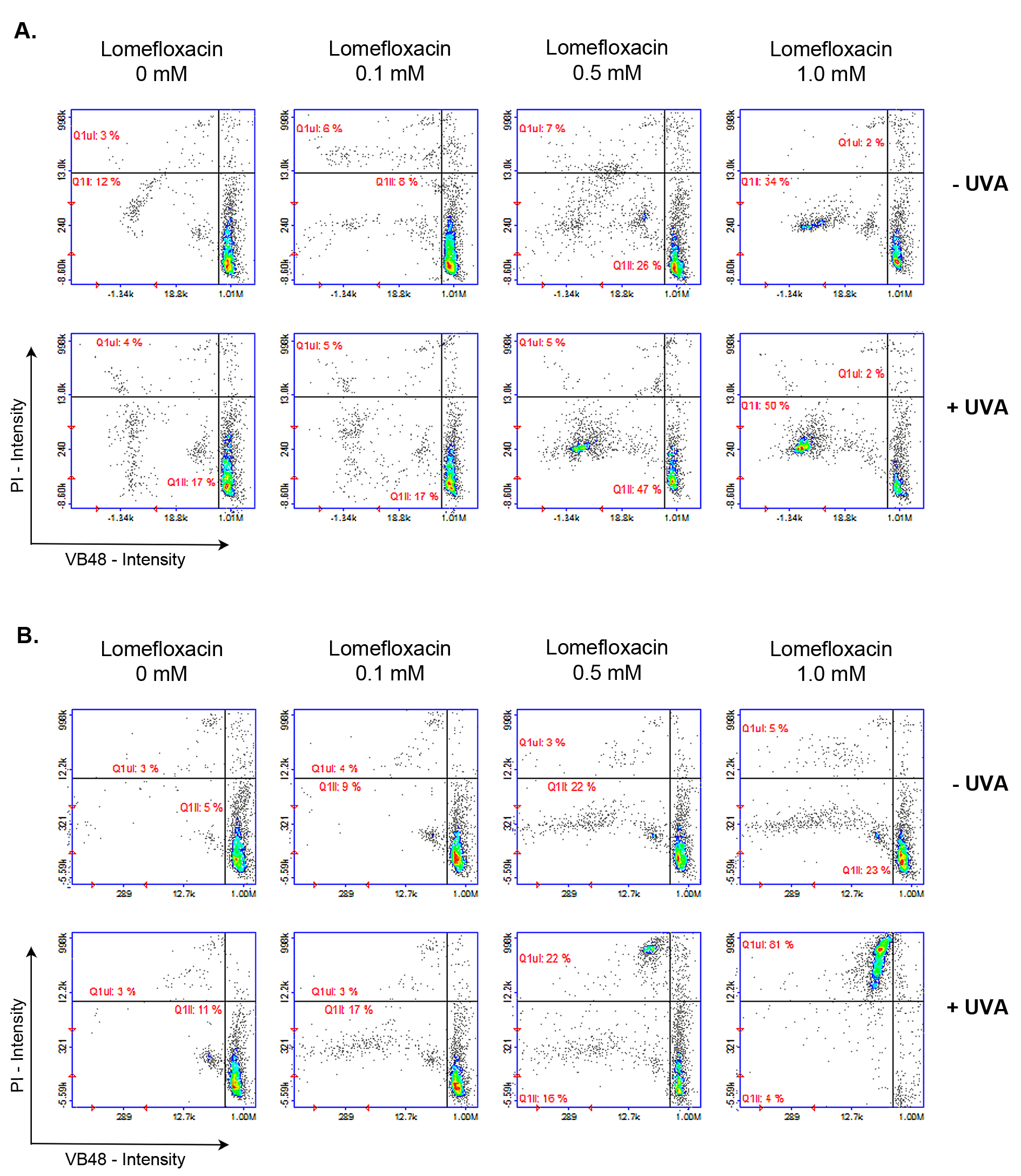

Supplement: Supplementary file 1 [file ijms-21-08937-s001.zip › S 1 - GSH.png]

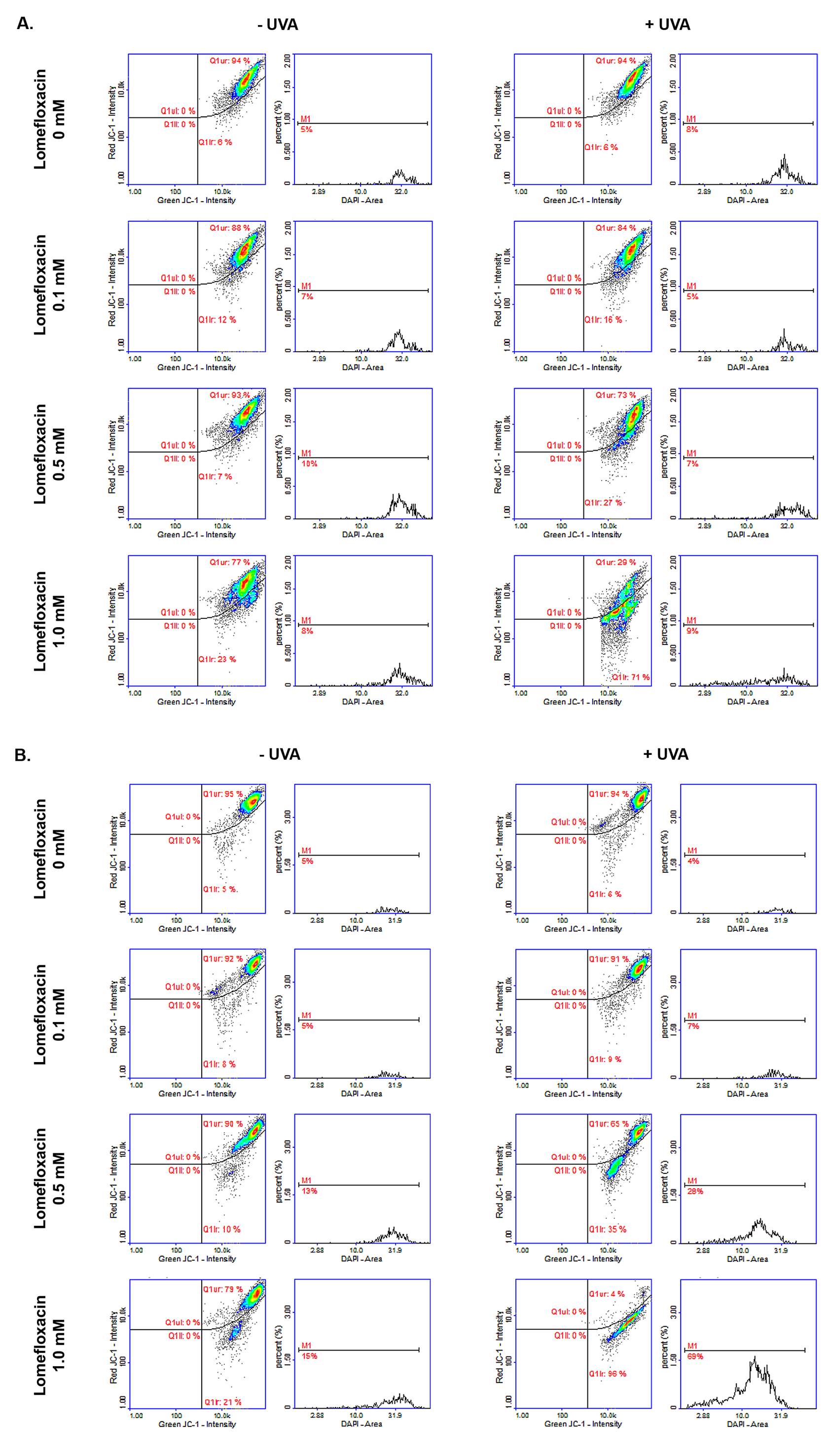

Supplement: Supplementary file 1 [file ijms-21-08937-s001.zip › S 2 - Mitochondrial potential.tif]

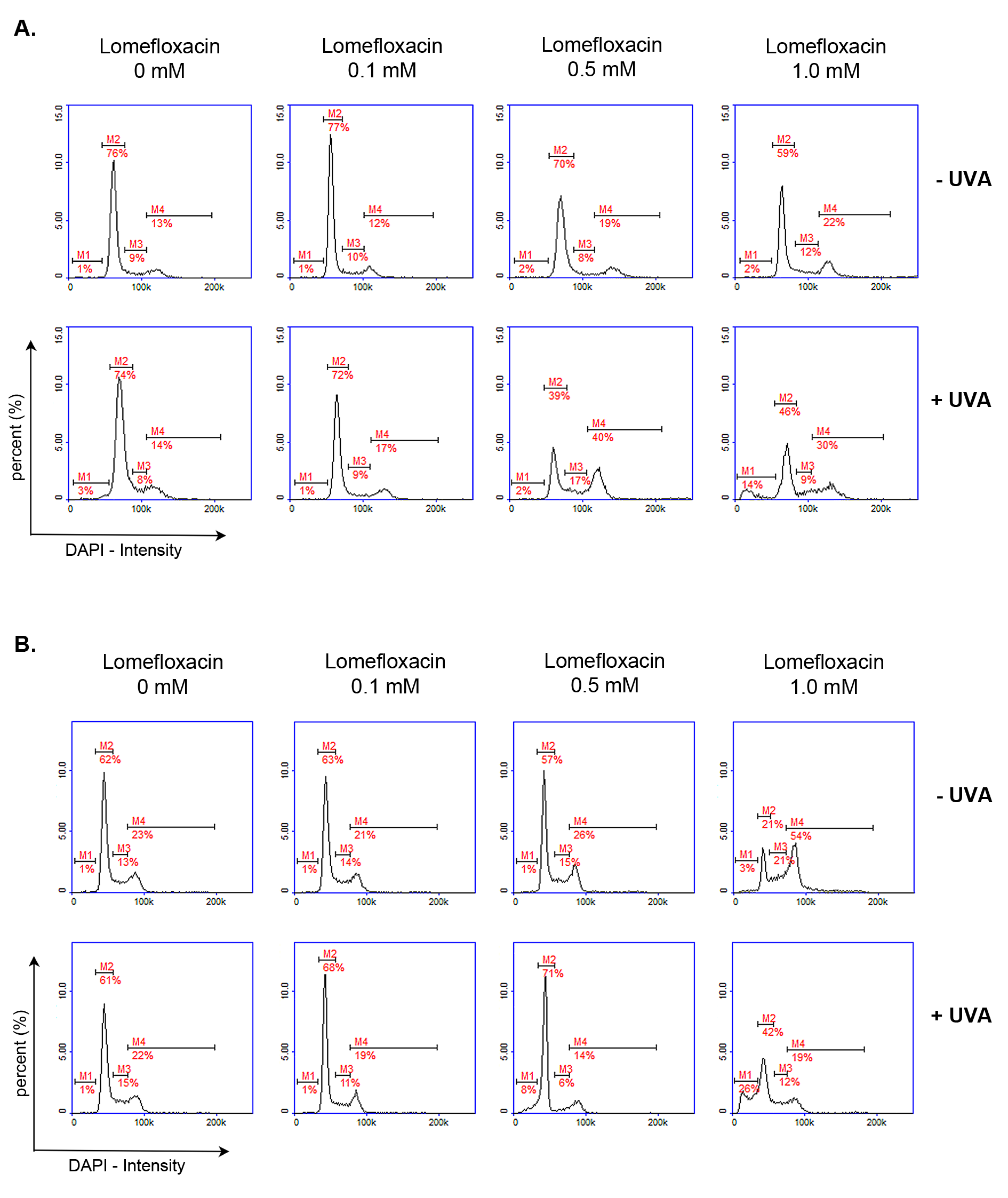

Supplement: Supplementary file 1 [file ijms-21-08937-s001.zip › S 3 - cell cycle.png]

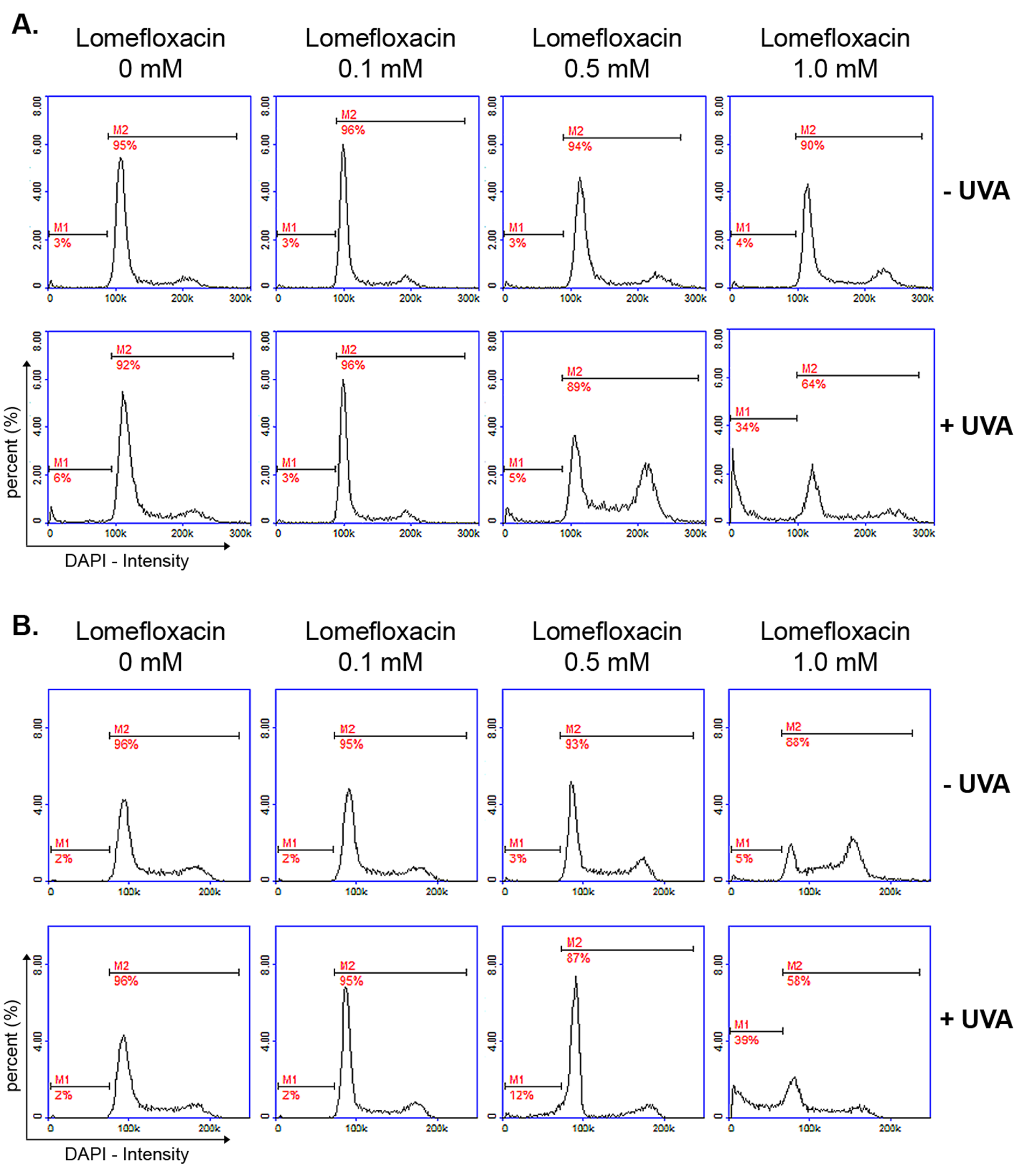

Supplement: Supplementary file 1 [file ijms-21-08937-s001.zip › S 4 - DNA fragmentation.png]
